# Genome and Comparative Transcriptome Dissection Provide Insights Into Molecular Mechanisms of Sclerotium Formation in Culinary-Medicinal Mushroom *Pleurotus tuber-regium*

**DOI:** 10.3389/fmicb.2021.815954

**Published:** 2022-02-17

**Authors:** Xueyan Sun, Junyue Wu, Shuhui Zhang, Lu Luo, Cuiyuan Mo, Li Sheng, Aimin Ma

**Affiliations:** ^1^College of Food Science and Technology, Huazhong Agricultural University, Wuhan, China; ^2^Key Laboratory of Agro-Microbial Resources and Utilization, Ministry of Agriculture, Wuhan, China

**Keywords:** *Pleurotus tuber-regium*, whole genome sequencing, RNA-seq, molecular mechanisms, sclerotium formation

## Abstract

*Pleurotus tuber-regium* is an edible and medicinal sclerotium-producing mushroom. The sclerotia of this mushroom also serve as food and folk medicine. Based on the description of its monokaryon genome, sequenced with Illumina and PacBio sequencing technologies, comparative transcriptomic analysis using RNA sequencing (RNA-seq) was employed to study its mechanism of sclerotium formation. The *de novo* assembled genome is 35.82 Mb in size with a N50 scaffold size of 4.29 Mb and encodes 12,173 putative proteins. Expression analysis demonstrated that 1,146 and 1,249 genes were upregulated and downregulated with the formation of sclerotia, respectively. The differentially expressed genes were associated with substrate decomposition, the oxidation-reduction process, cell wall synthesis, and other biological processes in *P. tuber-regium*. These genomic and transcriptomic resources provide useful information for the mechanism underlying sclerotium formation in *P. tuber-regium*.

## Introduction

Sclerotia of certain fungal species have important medicinal values and are popular in Chinese traditional medicine. A well-known, nutritious, and medicinal mushroom, *Pleurotus tuber-regium* (Fr.) Singer, also known as the tiger milk mushroom, produces sclerotia that also have a variety of edible and medicinal values, including serving as components of pork sausage; agents for thickening and flavoring soups; and remedies for ailments, such as headache, stomach pain, fever, and cold (Oso, 1977; Akobundu and Eluchie, 1992; Ohiri, 2018). Because of the health-promoting potential, research involving the sclerotia of *P. tuber-regium* focuses on the nutritive values, bioactive compounds, and clinical potential (Isikhuemhen et al., 2005; Wong and Cheung, 2008; Ohiri, 2018). However, omics studies to assess the molecular mechanisms underlying sclerotium formation in *P. tuber-regium* are lacking.

With the development of next-generation sequencing technology (Ansorge, 2009), omics technology provides a valid and comprehensive method for us to widely study the genetic basis in several sclerotium-producing mushrooms, such as *Wolfiporia cocos* (Zhang et al., 2016), *Lignosus rhinocerotis* (Yap et al., 2014), *Ophiocordyceps sinensis* (Li et al., 2016), and *Cordyceps guangdongensis* (Zhang C. et al., 2018). Genome and transcriptome dissection offer powerful and effective approaches that result in the identification and characterization of diverse genes in mushrooms related to metabolites, growth and development, and response to environments (Yap et al., 2014; Kramer and Nodwell, 2017; Gupta et al., 2018).

To acquire abundant information to understand molecular biological and genetic information related to sclerotium formation in *P. tuber-regium*, its genome was sequenced with Illumina and PacBio sequencing platforms. The genome features and annotation and the genome-wide distribution of non-coding RNAs (ncRNAs) and repetitive elements were stated intuitively. Some studies reveal that a series of functional genes that participate in sclerotial development are differentially expressed at early stages of sclerotium formation (Xing et al., 2013; Zhang et al., 2016). Thus, the newly formed sclerotia and vegetative mycelia of *P. tuber-regium* were used in transcriptome sequencing to authentically provide information for identifying functional genes regulating sclerotium formation. These genome and transcriptome resources supply new insights to understand the genetic basis and gene functions associated with this sclerotium-producing mushroom.

## Materials and Methods

### *Pleurotus tuber-regium* Strains and Culture Conditions

The *P. tuber-regium* strain ACCC 50657 was kept in the Laboratory of Food Microbiology, Huazhong Agricultural University. Monokaryotic strains 2 and 18 were a pair of compatible single-spore isolates from the fruiting body of *P. tuber-regium* strain ACCC 50657, and dikaryotic strain 218 was obtained by pairing the two monokaryotic strains. These strains were maintained and subcultured on solid potato dextrose agar (PDA) medium (potato 200 g/L, glucose 20 g/L, and agar 20 g/L) at room temperature and 32°C, respectively.

To induce formation of sclerotia, culture bags were prepared that were composed of cottonseed shell, bran, gypsum, lime, and H_2_O. These 300-g sterilized culture bags were inoculated with each *P. tuber-regium* strain and incubated at 32°C for 15 d to ensure complete colonization. Then, each culture bag was buried in 2.5 L of nutrient soil using a plastic vase (approximately 5.5 L) with a rounded bottom and incubated at room temperature with proper humidity.

### Genomic Analysis

Monokaryotic strain 18 was selected for genome sequencing. It was grown on PDA medium, covered with cellophane, and incubated at 32°C for 7 d in darkness. Genomic DNA was extracted using the CTAB extraction protocol (Cota-Sánchez et al., 2006). The DNA quality and quantity were determined using a Qubit dsDNA HS Assay Kit (Invitrogen, United States) and an Agilent Bioanalyzer 2100 (Agilent Technologies, United States).

After filtering low-quality reads, the genome survey was performed by counting the frequency of 17-mers from 5.7 Gb of data. Ten thousand randomly picked read pairs were blasted to the NCBI non-redundant nucleotide (nt) database to check for obvious sample contamination. Subsequently, 10 μg genomic DNA was used for 20 kb template library preparation using the BluePippin Size Selection system (Sage Science, United States) according to the manufacturer’s protocol (Pacific Biosciences, United States). The library was sequenced on the Pacific Biosciences Sequel platform. Assembly was performed using the Canu software with default parameters (Koren et al., 2017), and then, Pilon software was used to correct the preassembled structure according to NGS reads to obtain the final assembly result (Walker et al., 2014).

The protein-coding genes were annotated using predicted proteins to compare with seven major databases (NR, Swiss-Prot, KOG, KEGG, GO, Pfam, and TrEMBL), and the functional prediction of proteins was performed to combine the functional information of the proteins in the databases. Genes with putative CAZymes were annotated by BLASTP analysis according to the Carbohydrate Active Enzymes database^1^ (Cantarel et al., 2009).

There are two types of repetitive elements in the genome: tandem (e.g., microsatellites and minisatellites) and interspersed repeats (e.g., transposable elements or TEs). The presence of tandem repeats was investigated in all contigs using Tandem Repeats Finder (Benson, 1999). TE detection was performed with RepeatMasker^2^ based on the Repbase database^3^. The presence of snRNA and snoRNA was sought by aligning our genome assembly to the Rfam database^4^ using the *cmscan* program (Nawrocki and Eddy, 2013). tRNAs were analyzed using tRNAscan-SE (Chan and Lowe, 2019). rRNA was identified as combined Rfam with RNAMMER (Lagesen et al., 2007).

### Transcriptomic Analysis

Mycelia of dikaryotic strain 218 were grown on 60-g culture substrate resting in 250-mL glass beakers that had been covered with sterilized polyethylene film and incubated at 32°C. The aerial mycelia that climbed onto the beaker walls (10–14 days) were harvested. The sclerotia were harvested when they reached about 1 cm in diameter.

Total RNA was extracted from each mycelia and sclerotia sample using Trizol reagent, according to the manufacturer’s instructions (TaKaRa, Japan). The quality and quantity of extracted RNA were measured using 1.5% RNAase-free agarose gel electrophoresis, a NanoDrop 2000 spectrophotometer (NanoDrop Technologies, United States), and a Bioanalyzer 2100 system (Agilent Technologies, United States). The construction of cDNA library and RNA-seq of the eligible six RNA samples were carried out by Frasergen, Inc (Wuhan, China) using Illumina HiSeq sequencing.

The sequenced reads were aligned to our sequenced *P. tuber-regium* genome using Hisat2 (Kim et al., 2015) with default parameters. The aligned records from the aligners in the BAM/SAM format were further checked to remove potential duplicate molecules. Subsequently, gene expression levels were estimated using FPKM values (fragments per kilobase of exon per million fragments mapped) by Cufflinks software (Trapnell et al., 2010).

The EdgeR (v3.6.8) package method was used to evaluate differential gene expression between mycelial and sclerotial samples (Robinson et al., 2010). False discovery rate (FDR) values < 0.05 and | log_2_Fold Change| ≥ 1 were used as thresholds to assess the significance of gene expression differences.

Functional analysis of the transcriptome genes was carried out using GO (Ashburner et al., 2000; Young et al., 2010) and KEGG pathways (Kanehisa et al., 2004).

Reverse transcription quantitative polymerase chain reaction (RT-qPCR) was employed to analyze the eight upregulated and eight downregulated candidate genes among transcriptome samples to verify the quality of RNA-seq results. The gene-specific primers used for RT-qPCR are shown in Supplementary Table 1. The reactions were executed in QuantStudio™ 6 Flex System (Thermo Fisher Scientific, United States) using the following parameters: 95°C for 10 min, followed by 40 cycles of 95°C for 15 s, and 60°C for 1 min, and the melt curve for each product was detected. Three biological and three technical replicates were performed for each sample using the combination of *E3upl* and *Tif-5a* as the internal reference genes (Sun et al., 2021). The log_2_Fold Change of sclerotia relative to mycelia was calculated using the 2^–ΔΔ*Ct*^ method.

## Results

### Genome Features and Annotation

**FIGURE 1:**
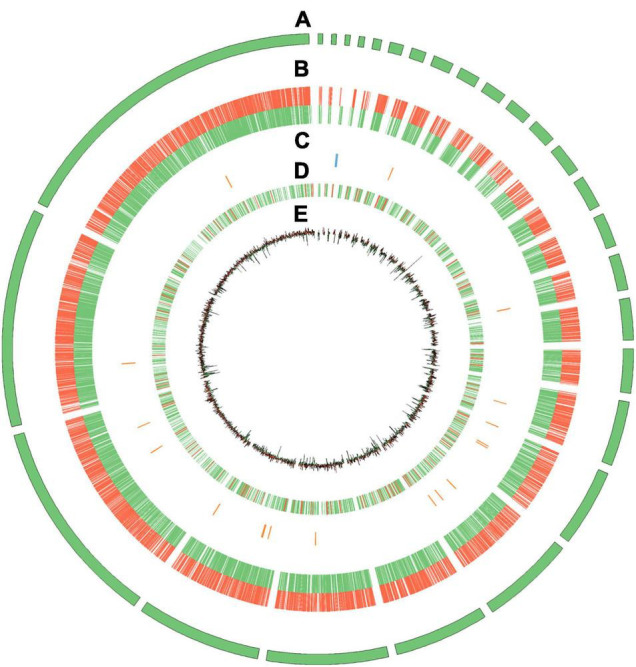
The general view of *P. tuber-regium* genome. **(A)** Contigs longer than 100 kb. **(B)** Gene density, represented as the number of genes in 100 kb non-overlapping windows. **(C)** Distribution of rRNA, tRNA, snRNA, and snoRNA. **(D)** Distribution of coverage of repetitive sequences. **(E)** GC content, increased in color from green to red.

In total, the final genome assembly of *P. tuber-regium* monokaryotic strain 18 yielded a total size of 35.82 Mb, 65 contigs, 47.5% G + C content, and a contig N50 of 4.29 Mb. Approximately, 527 × coverage data were acquired. The monokaryon genome is bigger in size than the dikaryon genome of this previously sequenced mushroom using the Illumina platform (Lam et al., 2018). The genome sequenced in our study is similar in size to the genomes of several other Agaricales species, including *L. rhinocerotis* (34.31 Mb) (Yap et al., 2014) and *P. ostreatus* (34.9 Mb) (Qu et al., 2016) but smaller than that of *W. cocos* (50.48 Mb) (Floudas et al., 2012), *P. tuoliensis* (48.2 Mb), and *P. eryngii* (49.9 Mb) (Zhang Z. et al., 2018). The general statistics and view of the sequenced *P. tuber-regium* genome are shown in Table 1 and Figure 1, respectively.

**TABLE 1:** Genome statistics of *P. tuber-regium*.

Contig features	
Total number	65
N50 (bp)	4292595
N90 (bp)	622912
Max length (bp)	6764344
Min length (bp)	512
Sequence GC content (%)	47.5
**Genome features**	
Genome assembly (Mb)	35.82
Number of protein-coding genes	12,173
Coding sequences/genome (%)	49.01
Average gene length (bp)	1442.14
Average exon length (nt)	228.81
Average intron length (nt)	66.45
Average number of exons per gene	5.11
CDS GC content (%)	49.96

A large portion of ncRNAs are functional and can produce various regulatory activities, such as signal molecules, ligands, and enzymes, and they are widely expressed in both prokaryotes and eukaryotes (Fu, 2014). The structural ncRNAs, including rRNA, tRNA, snRNA, and snoRNA, and regulatory ncRNAs were identified and mapped to the *P. tuber-regium* genome (Figure 1). Splicing catalysis by spliceosome is an essential step in the process of gene expression. The spliceosome is formed by the stepwise integration of five snRNPs composed of U1, U2, U4, U5, and U6 snRNAs and more than 150 proteins binding sequentially to pre-mRNA (Stark and Lührmann, 2006). Besides U1, the U2, U4, U5, and U6 snRNAs were also identified in our fungal strain. It is worth noting that U1 was also not identified in other basidiomycetous mushrooms, including *P. ostreatus* (Qu et al., 2016). With regard to tRNAs, 173 were annotated along with their loci, lengths, and transported amino acids. In addition, six snoRNAs, five 5s rRNAs, five 5.8s rRNAs, four 18s rRNAs, and four 28s rRNAs were identified in the genome.

Repetitive elements are essential for organismal development, including genome organization, evolution, epigenetic diversity, and plasticity (Foulongne-Oriol et al., 2013; Freire-Benéitez et al., 2016). Tandem repeats accounted for 1.13% of the genome assembly with an average length of 127 bp. The same as *Agaricus bisporus*, microsatellites appeared widely and distributed evenly over the whole genome sequence (Figure 1). The TEs, composed of Class I retrotransposons and Class II DNA transposons, accounted for 2.45% of the genome assembly with a total length of 876,249 bp. For class I, 339 sequences had homologies with long terminal repeats (LTRs), two sequences were observed having long interspersed nuclear elements (LINEs), and none had short interspersed elements (SINEs). For class II, 58 DNA transposons were identified in the genome assembly of *P. tuber-regium*.

### Transcriptome Sequencing, Assembly, and Functional Annotation

To better elucidate the molecular mechanisms of sclerotium formation in *P. tuber-regium*, RNA-seq was performed to compare gene expression differences between its mycelia and newly formed sclerotia. After data filtering and trimming, high-quality reads ranging from 29 to 37 Mb (mycelia) and 23 to 31 Mb (sclerotia) were obtained from the six examined libraries (Table 2). The Q30 of the six samples was more than 92%, indicating that the overall sequencing quality was satisfactory, and the data were suitable for further assembly analysis. Hisat2 software mapped over 89.47% of our clean reads to the genome, which was used for further analysis (Table 2). Annotation and functional classification results of 9,815 genes were obtained using the eight data sets. A total of 9,781 (77%) genes associated with known proteins in the Nr database, followed by TrEMBL database (9,769: 77%), Pfam database (6,623: 52%), SwissProt database (5,318: 41%), KOG database (4,264: 33%), GO database (4,210: 33%), KEGG database (2,922: 23%), and COG database (2,746: 21%).

**TABLE 2:** Transcriptome output and assembly data of mycelia and sclerotia samples in triplicate.

Results	Mycelium 1	Mycelium 2	Mycelium 3	Sclerotium 1	Sclerotium 2	Sclerotium 3
Clean reads	32204836	37000064	29067470	23574180	31104758	29760002
Clean reads pair	16102418	18500032	14533735	11787090	15552379	14880001
Clean reads Q30 (%)	93	93	92	93	93	93
Mapping	14707996	16906319	13276563	10597450	13915350	13527747
Unique	13453232	15534318	12257031	9811004	12461113	12501040
Mapping rate	91.34%	91.39%	91.35%	89.91%	89.47%	90.91%
Unique rate	83.55%	83.97%	84.34%	83.24%	80.12%	84.01%

**FIGURE 2:**
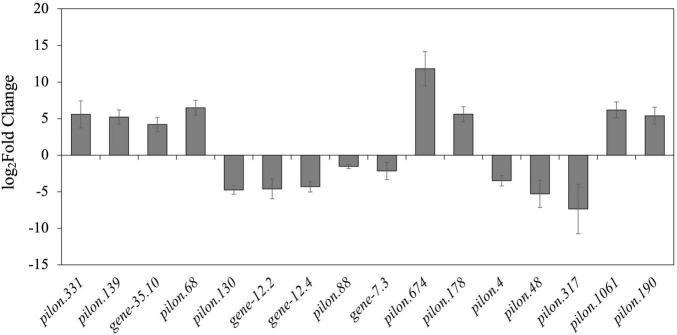
Bar chart based on RT-qPCR validation of observed expression levels for eight upregulated or downregulated DEGs. The log_2_Fold change values of the RT-qPCR assay were calculated by the 2^–ΔΔ*Ct*^ method and shown with standard deviations from the mean.

According to DEG analysis, we found conspicuous differences in the gene expression levels between mycelia and newly formed sclerotia. We compared libraries for gene expression differences that were ≥ 2 or ≤ −2 and detected 1,146 upregulated genes and 1,249 downregulated genes in the newly formed sclerotia compared with mycelia. To verify the reliability of the transcriptomic data, RT-qPCR of 16 DEGs that had been observed through RNA-seq showed that our profiling of these DEGs were consistent with RNA-seq results (Figure 2).

**FIGURE 3:**
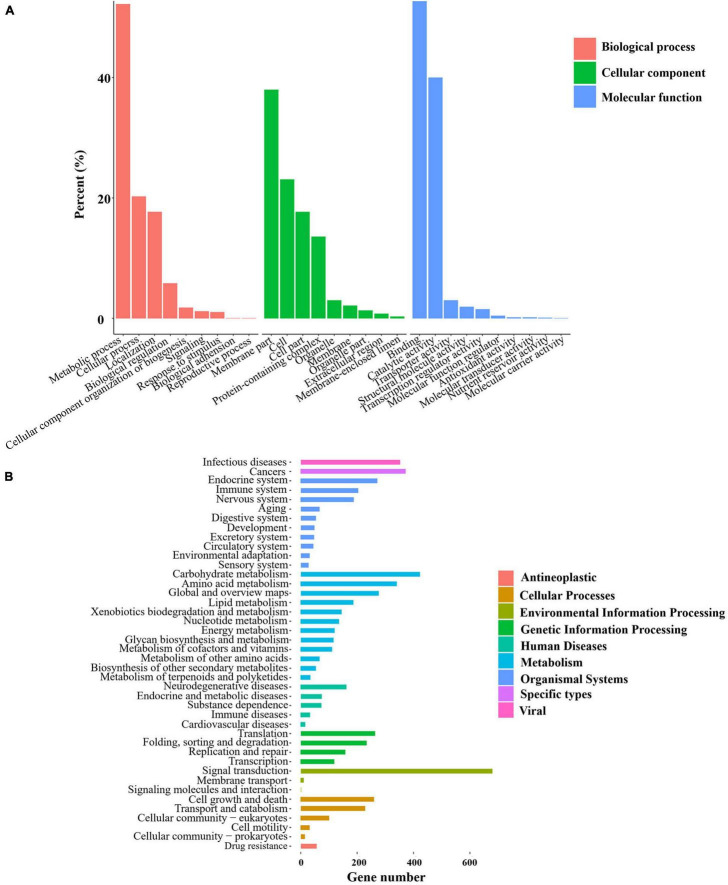
Statistics of GO and KEGG annotation results. **(A)** GO enrichment analysis of the *P. tuber-regium* transcriptome. The *Y*-axis indicates the percentage of a specific category. **(B)** KEGG enrichment analysis of pathways of the *P. tuber-regium* transcriptome. The X-axis indicates the number of genes in each function.

GO enrichment was conducted for the genes obtained from each group of samples, which were classified into the three primary GO categories annotated to biological process (GO-BP), cellular component (GO-CC), and molecular function (GO-MF) (Figure 3A). As shown from the results of GO-BP analysis, the most highly enriched term was metabolic processes, followed by cellular processes, illustrating that these processes are of functional importance for mycelial growth and sclerotial development in *P. tuber-regium*. In the category of GO-CC, membrane and cell were significantly enriched in the transcriptome genes of mycelia and sclerotia. In the GO-MF category, the most highly enriched term was binding. The second highly enriched GO-MF category was catalytic activity. Overall, the GO terms for the transcriptome genes of mycelia and sclerotia, which included metabolic process, membrane, and catalytic activity might potentially account for the formation of sclerotia in *P. tuber-regium*.

For better understanding of the functional pathways involved in sclerotia differentiation, we matched the transcriptome genes of mycelia and sclerotia to the KEGG database and displayed several metabolic and signal pathways (Figure 3B). We also conducted the KEGG enrichment analysis of DEGs, the KEGG results from our two groups of libraries revealed that DEGs listed in differentiation of mycelia to sclerotia were primarily enriched in the carbon metabolism; lysosome, cysteine, and methionine metabolism; and arginine and proline metabolism, etc (*p* < 0.05).

### Differentially Expressed Genes Related to Sclerotium Formation

Some studies revealed that a series of metabolic process participated in sclerotium formation (Yap et al., 2015; Zhang et al., 2016). The analysis of these DEGs mainly focused on carbohydrate metabolism, oxidation-reduction process, and cell wall synthesis. In addition, some gene clusters speculated to participate in the sclerotia development were further analyzed, such as carbohydrate active enzymes (CAZymes), oxidoreductases, chitin synthases, and hydrophobins.

**FIGURE 4:**
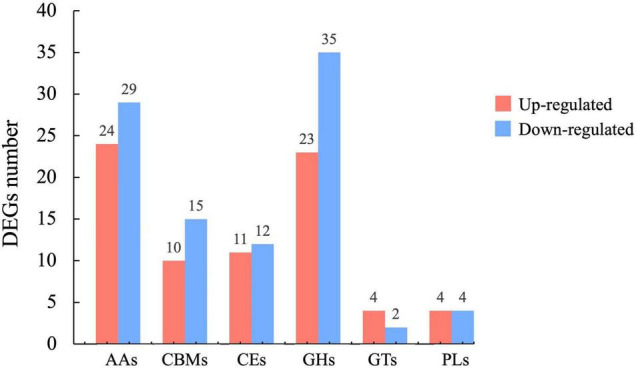
Histogram of upregulated (red bars) and downregulated (blue bars) CAZyme DEGs in newly formed sclerotia.

The differential expression of several genes in the CAZymes correlated well with our transcriptomic data. The CAZymes, including glycoside hydrolases (GHs), carbohydrate esterases (CEs), auxiliary activities (AAs), carbohydrate-binding modules (CBMs), glycosyl transferases (GTs), and polysaccharide lyases (PLs), are involved in carbohydrate metabolism. We analyzed the DEGs between mycelia and sclerotia and identified 76 and 97 CAZymes genes that were significantly upregulated and downregulated during the early stage of sclerotial development, respectively (Figure 4). Among these DEGs, more than half of them belonged to GH (33.53%) and AA (30.64%) families, indicating the importance of *P. tuber-regium* GHs and AAs families for sclerotium formation. In CAZymes, the CBMs encoded by *maker-contig_6_pilon-exonerate_protein2genome-gene-13.1* and *evm.TU.contig_17_pilon.35* were the most upregulated and downregulated in scletotia, respectively. It was reported that the CAZymes genes were not only involved in substrate decomposition, but also in sclerotium formation in *W. cocos* (Zhang et al., 2016). Our findings suggested that the CAZymes genes identified in *P. tuber-regium* might also participate in sclerotium formation.

We analyzed the D-glucose, the monomer of glucan and 1, 3-ß-glucan related pathway derived from the starch and sucrose metabolism pathway in KEGG. The beta-glucosidase genes (*evm.TU.contig_30_pilon.59* and *evm.TU.contig_5_pilon.88*) and glucan 1, 3-beta-glucosidase genes (*evm.TU.contig_3_pilon.1871* and *maker-contig_32_pilon-exonerate_protein2genome-gene-7.3*) involved in the D-glucose pathway were downregulated with the exception of gene *maker-contig_6_pilon-exonerate_protein2genome-gene-7.10*. This may account for the fact that sclerotia may only have early stage growth needs and typically exist in relatively dormant states during other growth periods when requirements of energy for life cycle activities are reduced. Additionally, with the exception of *evm.TU.contig_93_pilon.104*, most of the chitinase genes (*evm.TU.contig_13_pilon.236*, *evm.TU.contig_15_pilon.689*, and *evm.TU.contig_3_pilon.1375*) were downregulated in our *P. tuber-regium* sclerotia.

**FIGURE 5:**
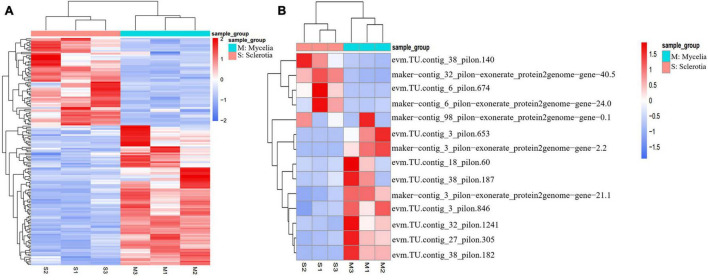
Heat map demonstration of expression patterns analysis in DEGs (*p* value < 0.05, | log_2_Fold Change| > 1) between sclerotia and mycelia using the FPKM. **(A)** Genes involved in oxidation-reduction progress show significant different expression in GO-BP. **(B)** Hydrophobins had more significant differential expression in sclerotia than in mycelia.

Genes related to the oxidation-reduction process were differentially expressed during sclerotium formation in *P. tuber-regium*. Oxidation -reduction–related genes were differentially expressed in mycelium vs. sclerotium production in other mushroom-producing species, such as *Polyporus umbellatus*, *W. cocos*, and *L. rhinocerotis* (Song et al., 2014; Yap et al., 2015; Wu et al., 2016; Li et al., 2017). The molecular mechanisms controlling sclerotium formation might include oxidative stress, signal transduction, and gene expression regulation (Sun et al., 2020). In this study, 155 genes involved in oxidation-reduction progress were significantly and differentially expressed in GO-BP (Figure 5A). Among them, 31 Cytochrome P450 (CYP) monooxygenases, demonstrating differential transcriptional abundances, deserved further attention. CYP monooxygenases have the unique ability to catalyze regio-, chemo-, and stereospecific oxidation of a wide range of substrates under mild reaction conditions, thereby addressing a significant challenge in chemocatalysis (Durairaj et al., 2016). Among the DEGs, the genes (*evm.TU.contig_17_pilon.68* and *evm.TU.contig_17_pilon.69*) encoding laccases were dramatically upregulated in sclerotia. Compared with the mycelia stage, the lipoxygenase encoded by *evm.TU.contig_25_pilon.317* in the sclerotia decreased significantly.

Differentially expressed genes involved in the cell wall system might participate in sclerotium formation in *P. tuber-regium*. Cell wall biogenesis is a very complicated process that incorporates not only multiple gene families directly involved in the polysaccharide biosynthesis and the remodeling of different cell wall components, but also structural proteins involved in cell wall assembly and rearrangement (Hu et al., 2017). We further analyzed genes with upregulated or downregulated expression in the formation of sclerotia that associated with GO cell composition terms related to “wall.” Notably, 16 cell wall–associated genes were screened, and 14 of them were hydrophobins (HPs), the structural constituents of cell walls. HPs are surface-active proteins unique in fungal cells and have roles in fungal growth as structural components and in the interaction of fungi with their environment (Linder et al., 2005; Kulkarni et al., 2017). In *P. tuber-regium* sclerotia, *evm.TU.contig_38_pilon.140* had the highest expression abundance, with more than 50,000 FPKM (Figure 5B). In particular, expression level of HP gene *evm.TU.contig_6_pilon.674* in sclerotia was hundreds of times greater than in mycelia. It is reported that HPs can affect the cell wall composition and protect emergent structures against adverse environmental conditions (Wetter et al., 2000; Wösten, 2001). We also observed an upregulated PII-type proteinase homologous to *Lactococcus lactis* subsp. *Cremoris* and a downregulated minor extracellular protease vpr homologous to *Bacillus subtilis* (strain 168) related to serine-type endopeptidase activity. Cell walls of sclerotia were thicker than those of mycelia in *P. tuber-regium* (Sun et al., 2020). This is likely because of the accumulation of cell wall components in addition to HPs, such as chitin, glycan, and xylan, as reported for *P. umbellatus* sclerotia (Li et al., 2021).

## Discussion

The interpretation of monokaryon genome and transcriptome information permitted us to research the gene function and biogenesis of sclerotia in *P. tuber-regium*. Compared with the early published genome, the improvements in sequencing and analysis methodology were reflected in the lower contig numbers and higher N50 length of the monokaryon genome. In the current study, genome sequencing results demonstrated fewer scaffolds (65), more protein-coding genes (12,173), and a longer N50 size (4.29 Mb). In KOG analysis, the three highest KOG annotations in descending order were general function prediction only; posttranslational modification, protein turnover, chaperones; and signal transduction mechanisms. In terms of KEGG analysis, the pathway was primarily enriched in signal transduction followed by carbohydrate metabolism. In the analysis of CAZymes, more CAZymes genes (776) were acquired compared with the published genome. They were composed of 202 AAs, 82 CBMs, 103 CEs, 251 GHs, 110 GTs, and 28 PLs. In addition to the different sequencing and analysis methods, the reasons for these differences might also be caused by the different sources of *P. tuber-regium* strains and the differences in monokaryon and dikaryon strains. Dikaryon not only added a complete and different set of genomes, but also interfered with each other between the two sets of monokaryon genomes, which affected the accuracy of sequencing results. Monokaryon could reduce the complexity of genome analysis. Monokaryotic genome sequencing provided advantages for the analysis of genes related to sclerotium formation and other biological characteristics without duplications. In other mushrooms, monokaryons were also used for genome sequencing, such as *Postia (Rhodonia) placenta*, *Dichomitus squalens*, and *Hericium erinaceus* (Gaskell et al., 2017; López et al., 2019; Gong et al., 2020). The high-quality monokaryon genome sequences of *P. tuber-regium* ACCC 50657-18 were used in this study to mine genes involved in the sclerotium formation. For example, genes coding CAZymes, oxidoreductases, and hydrophobins were analyzed. The actual functions of these genes during sclerotium formation in *P. tuber-regium* is still unknown though they are shown to play important roles in other sclerotium-producing mushrooms.

**FIGURE 6:**
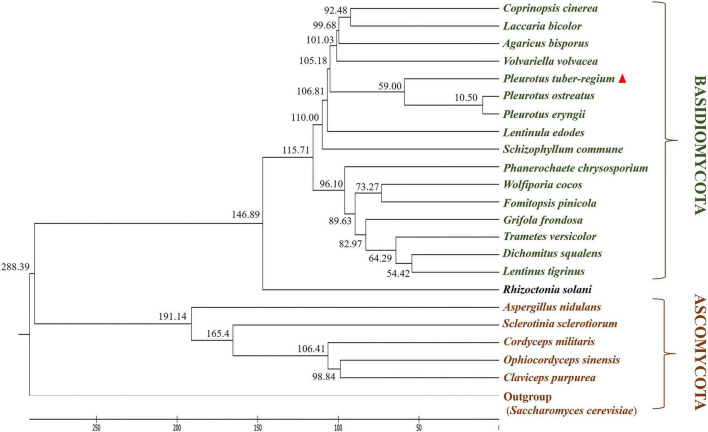
Phylogenetic tree of *P. tuber-regium* demonstrating the evolutionary distance with other fungal species. Time scale along the bottom indicates millions of years ago. The inferred divergence time of fungal species is located on the left side of each node.

Orthology is used to accurately describe differences and similarities in the composition of genomes among different species because orthologs share an ancestral gene that was present in a common ancestor of the compared species (Gabaldón and Koonin, 2013). Ortholog analysis was conducted for *P. tuber-regium* and the 22 other fungal species. A total of 50 single-copy orthologous genes were identified and were sequentially used to infer a phylogenetic tree (Figure 6) based on the neighbor-joining (NJ) method. Phylogenetic analysis revealed a close evolutionary relationship of *P. tuber-regium* to *P. ostreatus* and *P. eryngii*, supporting the classification of this fungus in the genus *Pleurotus* rather than *Lentinus* (Isikhuemhen et al., 2005). It should be noted that, although *P. tuber-regium* shares the same genus with *P. ostreatus* and *P. eryngii*, it is relatively distant from them and exhibits distinct physiological features, such as producing sclerotia. In addition, unlike other *Pleurotus* mushrooms, *P. tuber-regium* is a tropical mushroom and can grow in equatorial areas (Isikhuemhen et al., 2005; Wong and Cheung, 2008). *P. tuber-regium* also represents a unique intersterility group in the genus *Pleurotus* (Isikhuemhen et al., 2000).

Although the ability of *P. tuber-regium* to produce sclerotia was well-known, the potential molecular mechanisms and related functional genes in sclerotium formation were unknown. In this study, sclerotium morphogenesis was researched using a cotton seed hull cultivation substrate assay and constructed two libraries to acquire the DEGs and pathways related to sclerotium formation using high-throughput RNA-seq.

Mitogen-activated protein kinases (MAPKs) are key mediators of signaling in fungi, participating in the response to diverse stresses and developmental processes (Liu D. et al., 2019). We screened MAPK signaling pathway - yeast genes and observed upregulated expression of a catalase gene (*evm.TU.contig_3_pilon.1115*) and downregulated expression of Pheromone B alpha 3 receptor (*evm.TU.contig_25_pilon.509*) and GTP-binding protein rhoA (*evm.TU.contig_32_pilon.1268*) genes, and so on. CAZymes degrade the culture matrix to make the material available to fungi. Sixty-nine CAZymes genes were significantly upregulated during the early stage of sclerotial development compared with that of mycelia in *W. cocos* (Zhang et al., 2016). The CAZyme transcripts showed significant differences at the three stages of sclerotium development in *Morchella importuna* (Liu W. et al., 2019). In terms of oxidative stress response, mutants generated by deletion of *AflrsmA* in *Aspergillus flavus* displayed less sensitivity to the oxidative reagent tert-Butyl hydroperoxide and decreased production of sclerotia (Wang et al., 2020). Deletion of a sclerotial regulator in *A. niger*, *ansclR*, increased its susceptibility to oxidative stress (Lv et al., 2015). Antioxidant activity associated with glycolysis was critical for sclerotia growth in *P. umbellatus* (Li et al., 2017). DEGs were also enriched in oxidative stress–related genes and pathways, so these genes might play key roles in sclerotial biogenesis in *P. tuber-regium*. As for cell wall proteins, they play vital roles in different morphological stages, including mycelium, fruiting body, and sclerotium production in mushrooms (Chen et al., 2012). Although the *P. tuber-regium* sclerotia cell wall had high levels of protein content, the number of its identified cell wall proteins was much lower than those found in mycelia or fruiting bodies (Chen et al., 2012). Additionally, the sclerotial cell wall was significantly thicker than that of the mycelial cell wall in *P. tuber-regium* (Sun et al., 2020). Thus, DEGs associated with cell wall proteins deserve further verification and functional research.

The vegetative mycelia and newly formed sclerotia of *P. tuber-regium* were subjected to transcriptome sequencing analysis and assembly with reference genome, resulting in the first comprehensive transcriptome analyses related to sclerotium formation in *P. tuber-regium*. We screened DEGs and pathways associated with the MAPK signaling pathway, carbohydrate catabolism, cell wall system, oxidative stress response, and so on. Further studies are necessary to individually test the DEGs related to sclerotia biogenesis to better assess their developmental roles. In summary, the elucidation of genome and transcriptome data of *P. tuber-regium* in this manuscript provide a new reference for further studies of sclerotium formation.

## Accession Number

The genome sequence has been deposited at NCBI (accession number JACFYU000000000) with a BioProject number (PRJNA644065) and a BioSample number (SAMN15447141). The raw sequencing data of transcriptome were deposited at the NCBI Sequence Read Archive under Bioproject No. PRJNA801849.

## Data Availability Statement

The datasets presented in this study can be found in online repositories. The names of the repository/repositories and accession number(s) can be found in the article/Supplementary Material.

## Author Contributions

XS and AM designed the research. XS performed the experiments, analyzed the data, and wrote the manuscript. JW, SZ, LL, CM, LS, and AM critically reviewed the manuscript. All authors contributed to the article and approved the submitted version.

## Conflict of Interest

The authors declare that the research was conducted in the absence of any commercial or financial relationships that could be construed as a potential conflict of interest.

## Publisher’s Note

All claims expressed in this article are solely those of the authors and do not necessarily represent those of their affiliated organizations, or those of the publisher, the editors and the reviewers. Any product that may be evaluated in this article, or claim that may be made by its manufacturer, is not guaranteed or endorsed by the publisher.

## Footnotes

1
http://www.cazy.org

2
http://repeatmasker.org

3
http://www.girinst.org/repbase/

4
http://rfam.xfam.org/
