# Supplementary material for: Genome and Comparative Transcriptome Dissection Provide Insights Into Molecular Mechanisms of Sclerotium Formation in Culinary-Medicinal Mushroom Pleurotus tuber-regium
Source: Front Microbiol. 2022 Feb 17;12:815954. doi: 10.3389/fmicb.2021.815954 (PMC8891965; doi:10.3389/fmicb.2021.815954)
Supplement: Supplementary file 1 [file Table_1.DOCX]

**Supplementary Table 1** Primers for RT-qPCR amplification in this work.

| **Primers** | **Sequence (5‘ → 3’)** |
| --- | --- |
| evm.TU.contig_32_pilon.331-F | GGATCTCCGCCTCAGAATGG |
| evm.TU.contig_32_pilon.331-R | GGCACGCCTCCTTTCGTATA |
| evm.TU.contig_32_pilon.139-F | TGCACGGTGCTTCGTTCAT |
| evm.TU.contig_32_pilon.139-F | AAGTCTGCAAGGTCGGTGT |
| maker-contig_32_pilon-exonerate_protein2genome-gene-35.10-F | CAACGTACCCAACTTCCCGA |
| maker-contig_32_pilon-exonerate_protein2genome-gene-35.10-R | ATTCGACCGGCCTTCCAATT |
| evm.TU.contig_17_pilon.68-F | TTCGCTGTGAACGTCGAGAA |
| evm.TU.contig_17_pilon.68-R | CGCTCCAAGGTGTATACGCT |
| evm.TU.contig_12_pilon.130-F | ACTTCTGTAGTCAAGGCGGC |
| evm.TU.contig_12_pilon.130-R | ACCAGAACAGGGCATACTGC |
| maker-contig_6_pilon-exonerate_protein2genome-gene-12.2-F | ATGACCCGAACGCATCAACT |
| maker-contig_6_pilon-exonerate_protein2genome-gene-12.2-R | GTCGCTGTTCGGGTTTGAAC |
| maker-contig_6_pilon-exonerate_protein2genome-gene-12.4-F | GCAAAGAATGGTGCCTTGGG |
| maker-contig_6_pilon-exonerate_protein2genome-gene-12.4-R | ATTGATTCGAGCGGGGTTGT |
| evm.TU.contig_5_pilon.88-F | GCTTGCTTCTCTCCCGTTCT |
| evm.TU.contig_5_pilon.88-R | TTGTCAGCCCATGAGCCATT |
| maker-contig_32_pilon-exonerate_protein2genome-gene-7.3-F | ACGAAGTACACCCCGCAAAT |
| maker-contig_32_pilon-exonerate_protein2genome-gene-7.3-R | GGACCAAGCGGGAGATTCAA |
| evm.TU.contig_6_pilon.674-F | TATTGCAAGGTGCCACCGT |
| evm.TU.contig_6_pilon.674-R | ACCAACAGCAATGACGCCA |
| evm.TU.contig_5_pilon.178-F | TGGACGTGTTTGCAAGCCT |
| evm.TU.contig_5_pilon.178-R | ATCCAGCAACGGCCATCTT |
| evm.TU.contig_78_pilon.4-F | AAATCCGCGGGGTCGAATT |
| evm.TU.contig_78_pilon.4-R | ATGAGCCAGATGCCATGGT |
| evm.TU.contig_3_pilon.48-F | ACTCAGTGTTCGACATGCGT |
| evm.TU.contig_3_pilon.48-R | TTCCTTGACTGCACGCCAT |
| evm.TU.contig_25_pilon.317-F | ATGATGCGGCAGGTTAAGC |
| evm.TU.contig_25_pilon.317-R | TCGCAACATCGAAGAGCCA |
| evm.TU.contig_3_pilon.1061-F | ACTGCTGCCGATGTCGATT |
| evm.TU.contig_3_pilon.1061-R | AGACGTGCAAGGCCTGAAA |
| evm.TU.contig_6_pilon.190-F | AATGTCGACCAGCTGAAGCA |
| evm.TU.contig_6_pilon.190-R | GAGCTTGCGTTGTTGCTGAA |
